# Yoga intervention on the fatigue-pain-sleep disturbance symptom cluster for breast cancer patients receiving adjuvant chemotherapy: a pilot randomized controlled trial

**DOI:** 10.1186/s12906-026-05285-7

**Published:** 2026-02-10

**Authors:** Yishu Qi, Ying Cao, Yanhong Deng, Xing Ma, Dorothy Ngo Sheung Chan, Cho Lee Wong, Yao Guo

**Affiliations:** 1 The Nethersole School of Nursing, Faculty of Medicine, The Chinese University of Hong Kong, Shatin, N.T., R824 Esther Lee Building, Shatin, N.T., Hong Kong SAR, China; 2 School of Nursing, Xinjiang Medical University, Xinjiang Urumqi, China; 3 Breast Disease Center, The First Affiliated Hospital of Nanchang University, Nanchang, Jiangxi China

**Keywords:** Breast cancer, Yoga, Chemotherapy, Symptom management, Pilot study, Randomized controlled trial

## Abstract

**Background:**

The fatigue-pain-sleep disturbance symptom cluster is a common and notable issue negatively impacting breast cancer patients receiving adjuvant chemotherapy. Yoga is a promising strategy for symptom cluster management. This study aimed to examine the feasibility, acceptability, and preliminary effects of yoga intervention on the fatigue-pain-sleep disturbance symptom cluster in breast cancer patients receiving adjuvant chemotherapy to explore its effect on alleviating patients’ symptom burden.

**Methods:**

In a parallel-group pilot randomized controlled trial, 30 participants were randomly assigned to the intervention group or the control group. The participants in the control group received usual care, while those in the intervention group received yoga intervention and usual care. Feasibility was assessed using eligibility rate, recruitment rate, retention rate, adherence rate, and session attendance rate. Acceptability was assessed using a satisfaction scale and open-ended questions. The feasibility and acceptability of the intervention were evaluated post-intervention. Preliminary effects were assessed at baseline, post-intervention, and one-month follow-up.

**Results:**

Thirty participants were recruited, with a recruitment rate of 55.6%. Two participants dropped out of the study, and ten participants in the intervention group completed all intervention sessions, with a retention rate of 93.3% and a session attendance rate of 91.4%, showing high feasibility. 92.9% of participants expressed total satisfaction with the intervention, showing high acceptability. The yoga intervention achieved statistically significant post-intervention effects on the symptom cluster severity (*P* = 0.042), depression (*P* = 0.045), and health-related quality of life (*P* = 0.006) in breast cancer patients receiving adjuvant chemotherapy. No adverse event was reported.

**Conclusions:**

This pilot study supports the feasibility and acceptability of yoga intervention for breast cancer patients receiving adjuvant chemotherapy. The results suggest that yoga intervention has potentially positive effects on the management of the fatigue-pain-sleep disturbance symptom cluster and related outcomes. Future research with a larger sample size is warranted to examine the long-term effects of yoga intervention.

**Trial registration:**

The study was registered on the Chinese Clinical Trial Registry (www.chictr.org.cn), and trial registration number ChiCTR2400081863. The registration date was 14/03/2024.

**Supplementary Information:**

The online version contains supplementary material available at 10.1186/s12906-026-05285-7.

## Background

Breast cancer has become the most frequently diagnosed malignancy among women and the second most common cancer worldwide [1]. During adjuvant chemotherapy, breast cancer patients commonly encounter a wide range of adverse symptoms, some of which are interrelated and form symptom clusters [2]. The fatigue-pain-sleep disturbance symptom cluster is the most prevalent throughout the cancer trajectory for breast cancer patients, particularly before and during treatment [3].

The fatigue-pain-sleep disturbance symptom cluster is frequently accompanied by psychological symptoms like anxiety, depression, and stress [3], which are closely interrelated and mutually influential. Previous studies have demonstrated that higher levels of anxiety, depression, and stress are associated with more severe fatigue, pain, and sleep disturbances; in turn, the worsening of these symptoms further exacerbates patients’ depression and stress levels [4–6]. Consequently, the impact of the symptom cluster is not merely the additive effect of individual symptoms but rather the synergistic interaction among multiple interrelated symptoms, which increases patients’ overall symptom burden, impedes functional recovery, and adversely affects their quality of life [7, 8].

Current management strategies for symptom clusters typically focus on individual symptoms. When multiple symptoms occur simultaneously, applying separate interventions for each symptom may complicate management, potentially weakening the effectiveness of management strategies and increasing the treatment burden [2]. Rather than merely aggregating individual-symptom interventions, effective management of the symptom cluster should integrate strategies that simultaneously address multiple related symptoms. This approach can simplify complex symptom management and enhance the applicability of individual interventions, making them beneficial for multiple related symptoms or symptom clusters [2]. For breast cancer patients, the management of symptom clusters that cover a broader range of symptoms can allow more comprehensive and effective symptom burden relief, advance functional recovery, and improve their quality of life.

Yoga represents a promising management strategy for the fatigue-pain-sleep disturbance symptom cluster. Emerging evidence suggests that inflammation may be a potential mechanism for the occurrence of this cluster [9]. Yoga not only regulates inflammatory factors through physical postures, potentially alleviating the symptom cluster [10], but also guides participants in breathing and meditation, focusing on specific postures with inner awareness [11, 12], which relieves stress and regulates emotional experiences such as anxiety and depression [13, 14], thereby mediating neuroendocrine pathways, downregulate inflammation [15], and ultimately potentially fostering the management of the fatigue-pain-sleep disturbance symptom cluster. The efficacy of yoga in alleviating individual symptoms within this cluster—fatigue [16], pain [17], and sleep disturbance [18]—has been demonstrated in breast cancer populations.

However, existing yoga interventions have not been shown to effectively manage all three symptoms simultaneously, as at least one symptom within this cluster fails to show significant improvement [19]. In other words, current yoga interventions remain insufficient for managing the fatigue-pain-sleep disturbance symptom cluster comprehensively. Further development of interventions tailored to the characteristics of this cluster and the target population is warranted to improve symptom management. Moreover, previous research seldom examined co-occurring psychological symptoms such as anxiety, depression, and stress. Consequently, whether yoga interventions can simultaneously alleviate these interrelated psychological and physical symptoms remains unclear, limiting the broader application of yoga for comprehensive symptom management. As most yoga studies have been conducted in Western countries [19], their findings may not be generalized to Chinese breast cancer patients due to cultural background differences. Therefore, it is necessary to explore and confirm the feasibility and acceptability of yoga among Chinese breast cancer patients and to assess its preliminary effectiveness and broader applicability.

Accordingly, a yoga intervention was designed for breast cancer patients receiving adjuvant chemotherapy to evaluate its feasibility and acceptability and to examine its preliminary effects on the fatigue-pain-sleep disturbance symptom cluster, as well as anxiety, depression, stress, functional status, and quality of life in this population, thereby exploring its potential impact on patients’ symptom burden.

## Methods

### Study design

This study was a prospective, two-armed, single-blinded, pilot randomized controlled trial in accordance with the CONSORT Statement [20] (Supplementary Material 1).

### Ethical approval and registration

This study has received ethical approvals from the Joint Clinical Research Ethics Committee of the New Territories East Cluster of the Chinese University of Hong Kong and the research hospital in Mainland China. This study was conducted in accordance with the Declaration of Helsinki and has been registered: ChiCTR2400081863.

### Participants

This study was conducted at a tertiary comprehensive hospital in Jiangxi Province, China. The inclusion criteria were patients: (1) were diagnosed breast cancer and aged 18 years or older; (2) had completed surgery and were scheduled to continue with at least eight weeks of adjuvant chemotherapy; (3) could understand Chinese and follow instructions; (4) had reliable internet connectivity and access to internet devices; and (5) reported at least two symptoms within the target cluster, each rated ≥ 1 on a 0–10 numeric rating scale in the past 24 h, as assessed by the Chinese version of the MD Anderson Symptom Inventory (MDASI-C). Exclusion criteria for participants included: (1) diagnosis of metastatic breast cancer; (2) a concurrent diagnosis of other malignancies; (3) mental illness documented in medical records that might interrupt the completion of treatment in their medical records; or (4) currently participating in other exercise interventions. The eligible participants were recruited from March 18 to April 20, 2024.

### Study sample

A minimum of 12 samples per group is recommended in the pilot study [21]. Therefore, we set the sample size for this pilot study to at least 12 participants per group, with a total of at least 24 participants.

### Recruitment, randomization, and allocation

The principal investigator and a clinical nurse from the department assessed the eligibility of potential participants and invited qualified individuals to participate in the study. After informed consent was obtained and baseline data were collected, participants were randomly assigned to the intervention and control groups at a 1:1 ratio using a permuted block design (randomly variable block sizes of 4 and 6). Randomization was performed by a research assistant who was not involved in participant recruitment, intervention implementation, or data collection, using the computer-generated online randomization program (https://www.sealede nvelope.com/). This blinded research assistant conducted the allocation concealment by using opaque sealed envelopes.

### Intervention

The yoga intervention was developed based on our team’s previous findings [19], yoga theory [22, 23], and existing evidence concerning symptoms within the symptom cluster [18, 24, 25]. It integrated evidence from studies involving breast cancer patients receiving adjuvant chemotherapy to ensure that the program was tailored to their practical needs and preferences while remaining consistent with the guiding principles of yoga theory. Our previous systematic review demonstrated that high-frequency and long-duration interventions, and a delivery mode combining in-person and home-based sessions achieved favorable outcomes in managing the fatigue-pain-sleep disturbance symptom cluster in breast cancer patients receiving chemotherapy [19]. Furthermore, prior evidence indicated that eight weeks of 60-min yoga sessions, conducted two to three times per week, effectively alleviated fatigue [24], sleep disturbance [18], and pain [25] in breast cancer patients. In addition, previous research found that multicomponent yoga interventions incorporating elements such as breathing and postural practices effectively improved these symptoms, consistent with yoga theory [19, 22]. The yoga intervention was further refined through the qualitative interviews of 25 breast cancer patients receiving adjuvant chemotherapy to explore their exercise preferences. The results indicated that they preferred practicing in groups, under the guidance of video materials, and practicing in the early morning and evening. Additionally, the developed yoga intervention was commented on by an expert panel (*n* = 7) consisting of two oncology nurses, one oncology doctor, three yoga instructors, and one physiotherapist specializing in sports rehabilitation.

The yoga intervention encompassed in-person sessions and home-based sessions. When participants visited the hospital for chemotherapy, in-person sessions were performed in the activity room of the department by the principal investigator, who has oncology nursing experience and is also a certified yoga instructor. Each in-person session involved two to eight participants. During intervals between chemotherapy cycles, participants attended group-based supervised online meetings, which were conducted via Tencent Meeting software, for home-based sessions. During online meetings for home-based sessions, participants followed the yoga videos for practice. The principal investigator attended each online meeting for provide supervision. Each in-person or home-based session lasted 60 min, consisting 10 min of breathing practice, 35 min of posture practice (warm-up postures, standing postures, sitting postures, and relaxation postures), and 15 min of meditation. Each movement was specifically designed to address the unique needs of breast cancer patients receiving adjuvant chemotherapy, with particular consideration for postoperative upper limb functional recovery, symptom relief, disease burden, and physical activity limitation. The entire intervention lasted eight weeks, with three sessions per week. The yoga intervention was designed to be gentle, incorporating multiple breathing regulation between postures to help participants restore physical strength, and encouraging participants to focus on their own movements and progress rather than the precise accuracy of standard poses. Furthermore, the yoga intervention was progressive, starting with a basic program for the first four weeks and an advanced program in the subsequent four weeks to increase the difficulty and number of postures, enhancing effectiveness. Participants received a booklet detailing the yoga intervention contents, and researchers maintained weekly telephone contact with each participant to provide support and monitor progress.

### Control group

For the control group, participants underwent usual care, encompassing information about chemotherapy during hospitalization, medication guidance, health education (lymphedema prevention and infusion port management), and post-discharge reexamination recommendations.

### Treatment fidelity

To ensure consistency, all sessions in the yoga intervention were implemented by the principal investigator, a nurse with experience working in the oncology department who has been certified as a yoga instructor. Every session was recorded and randomly reviewed by the supervisor and a yoga instructor holding an RYT-500 certification with ten years of teaching experience.

### Outcome measures

#### Feasibility

The feasibility of the intervention was verified by calculating eligibility, recruitment, retention, adherence, and session attendance rates. The eligibility rate was calculated as the percentage of patients meeting the inclusion and exclusion criteria among those screened for eligibility. The recruitment rate was calculated as the percentage of patients who were successfully recruited among eligible patients. The retention rate was the percentage of participants persisting in completing the study out of all included participants. The adherence rate in the intervention group was the percentage of participants who completed all intervention sessions out of all participants in the intervention group. The session attendance rate was the percentage of the number of sessions attended by participants among the total number of sessions in the intervention group. Participants who were absent from a session or dropped out of the study and the reasons for their absence were all recorded. Any adverse event was also recorded.

#### Acceptability

The research team developed a 10-item questionnaire for this study to assess the acceptability of the intervention, using a 5-point Likert scale that ranged from 1 (‘strongly disagree’) to 5 (‘strongly agree’) (Supplementary Material 2). The questionnaire evaluated participants’ satisfaction with the intervention content and delivery, the number and duration of sessions, and their ability to independently complete the intervention. Open-ended questions were also included to obtain participants’ experiences and overall satisfaction with the yoga intervention.

#### The fatigue-pain-sleep disturbance symptom cluster

The fatigue-pain-sleep disturbance symptom cluster was evaluated using the Chinese version of the M.D. Anderson Symptom Inventory (MDASI-C). The severity of the symptom cluster was by calculating the average score of items about fatigue, pain, and sleep disturbance in MDASI-C. Each item was scored from 0 to 10, with 0 scores representing “not present” and 10 scores representing “as bad as you can imagine” within the past 24 h. The interference of the symptom cluster was assessed using the six interference items in MDASI-C, on a scale of 0–10, to reflect the interference degree of daily life of patients by the symptom cluster in the past 24 h. The MDASI-C has been widely used in Chinese breast cancer patients, with good internal consistency (Cronbach’s α = 0.83 −0.85) [26].

#### Anxiety and depression

Anxiety and depression were evaluated with the Chinese version of the Hospital Anxiety and Depression Scale (HADS), which constitutes seven items for anxiety and seven items for depression. Each item was rated on a four-point Likert scale, and higher scores are indicative of higher anxiety and depression levels. The Chinese version has good reliability and validity [27].

#### Stress

Stress was assessed with the Chinese version of the Perceived Stress Scale (PSS), which is made up of 10 items, among which items 4, 5, 7, and 8 are reverse scored. Each item is scored on a five-point scale ranging from 0 (never) to 4 (very often). PSS has good reliability and validity among Chinese breast cancer patients [28, 29].

#### Functional status

The Karnofsky Performance Scale (KPS) was used to assess the functional status of participants, which ranges from 0 to 100. A score of 0 indicates death, while a score of 100 represents normal function with no complaints or evidence of disease.

#### Health-related quality of life

Health-related quality of life was the Functional Assessment of Cancer Therapy-Breast (FACT-B) Chinese Version, a 37-item scale designed specifically to assess breast cancer patients. Higher scores reflect higher level of quality of life. The Chinese version has high validity and reliability [30].

### Data collection

Data were collected at three time points: baseline (T0), immediately after the intervention (T1), and one month post-intervention (T2), by a research assistant who was blinded to group allocation. Paper questionnaires were used to collect all outcomes at T0 and T1, whereas electronic questionnaires were administered at T2. Participants were encouraged to complete the questionnaires independently. Assistance was provided only when necessary, such as offering neutral explanations if participants found any items in the questionnaire confusing. At T1, participants in the intervention group were asked by the principal investigator about their acceptability of the intervention.

### Data analysis

Data analysis was conducted by a blinded statistician using IBM SPSS Statistics (version 27.0) as per the intention-to-treat principle [31], all randomized participants were analyzed according to their originally assigned groups. Outcomes were analyzed using generalized estimating equations (GEE), which incorporate all available repeated measurements contributed by each participant over time. Accordingly, participants who discontinued the intervention or follow-up remained in the analysis and contributed their observed data up to the time of discontinuation. Continuous variables were compared using the independent *t*-test and categorical variables were compared using the Fisher exact test or Mann–Whitney U test to determine significant differences in sociodemographic and disease-related characteristics between the two groups. The GEE model was employed to identify changes across groups and time points, including the severity and interference of the symptom cluster, anxiety, depression, stress, functional status, and health-related quality of life. The GEE model can be used since it can analyze repeated data for different types of outcomes to assess the overall effect of treatment over time [32]. Two-sided *p* < 0.05 represented a statistically significant difference. Hedge’s g was used to estimate the effect size, with estimates over 0.2, 0.5, and 0.8 reported as small, medium, and large effects [33].

## Results

### Characteristics of participants

The research team developed a socio-demographic sheet for this study to collect participants’ characteristics (Supplementary Material 2). The results showed that the mean age of all participants was 46.60 years (standard deviation (SD): 8.41). Most participants were married (83.3%), lived with their spouse or children (83.3%), and used medical insurance to cover treatment costs (90%). Approximately half of the participants lived in urban areas, had a junior high school education, were employed but on medical leave, and reported a monthly household income of less than 6,000 yuan. In terms of disease-related characteristics, most participants were at cancer stages I − II and had undergone modified radical mastectomy and sentinel lymph node biopsy. No significant differences in demographic characteristics were observed between the two groups at baseline. The characteristics of 30 participants are detailed in Table 1.

**Table 1:** Characteristics of the participants

Characteristics	All (*n* = 30)	Yoga group (*n* = 15)	Control group (*n* = 15)	*P*
Age (years), mean (SD)	46.40 (8.41)	43.86 (9.69)	48.93 (6.23)	0.102^a^
Marital Status				1.000^b^
Married	25 (83.3%)	12 (80%)	13 (86.7%)	
Unmarried	2 (6.67%)	1 (6.7%)	1 (6.7%)	
Divorced	3 (10%)	2 (13.3%)	1 (6.7%)	
Residence				
Rural	13 (43.3%)	7 (46.7%)	6 (40%)	1.000^b^
Urban	17 (56.7%)	8 (53.3%)	9 (60%)	
Mode of residence				
Living alone	2 (6.67%)	1 (6.7%)	1 (6.7%)	1.000^b^
Living with spouse(s) or children	25 (83.3%)	12 (80%)	13 (86.7%)	
Living with parents	3 (10%)	2 (13.3%)	1 (6.7%)	
Education				0.838^c^
Primary school	3 (10%)	1 (6.7%)	2 (13.3%)	
Junior high school	12 (40%)	6 (40%)	6 (40%)	
Senior high school/Technical secondary school	8 (26.7%)	5 (33.3%)	3 (20%)	
Bachelor’s degree	7 (23.3%)	3 (20%)	4 (26.7%)	
Employment status				0.474^b^
On the job	2 (6.7%)	2 (13.3%)	0 (0%)	
On sick leave	13 (43.3%)	8 (53.3%)	5 (33.3%)	
Self-employed	3 (10%)	1 (6.7%)	2 (13.3%)	
Unemployed	9 (30%)	3 (20%)	6 (40%)	
Retired	3 (10%)	1 (6.7%)	2 (13.3%)	
Precipitate monthly household income				
**< **3000 RMB	7 (23.3%)	4 (26.7%)	3 (20%)	0.896^c^
3000–5999 RMB	12 (40%)	5 (33.3%)	7 (46.7%)	
6000–8999 RMB	8 (26.7%)	4 (26.7%)	4 (26.7%)	
**≥ **9000 RMB	3 (10%)	2 (13.3%)	1 (6.7%)	
Payment method for medical expenses				1.000^b^
Self-funded	3 (10%)	1 (6.7%)	2 (13.3%)	
Medical insurance	27 (90%)	14 (93.3%)	13 (86.7%)	
Cancer stage				
Ⅰ	2 (6.7%)	0 (0%)	2 (13.3%)	0.392^c^
Ⅱ	19 (63.3%)	10 (66.7%)	9 (60%)	
III	9 (30%)	5 (33.3%)	4 (26.7%)	
Surgery				
Breast-conserving surgery	8 (26.7%)	4 (26.7%)	4 (26.7%)	1.000^b^
Modified radical mastectomy	22 (73.3%)	11 (73.3%)	11 (73.3%)	
ALND				
Yes	5 (16.7%)	2 (13.3%)	3 (20%)	1.000^b^
No	25 (83.3%)	13 (86.7%)	12 (80%)	
SLNB				1.000^b^
Yes	23 (76.7%)	11 (73.3%)	12 (80%)	
No	7 (23.3%)	4 (13.3%)	3 (20%)	
Chemotherapy regimens				0.868^b^
AC-T	18 (60%)	8 (53.3%)	10 (66.7%)	
EC-T	4 (13.3%)	2 (13.3%)	2 (13.3%)	
TcbHP	8 (26.7%)	5 (33.3%)	3 (20%)	
Current chemotherapy cycle				0.320^c^
Ⅰ	9 (30.0%)	5 (33.3%)	4 (26.7%)	
Ⅱ	7 (23.3%)	4 (26.7%)	3 (20.0%)	
III	5 (16.7%)	2 (13.3%)	3 (20.0%)	
IV	5 (16.7%)	3 (20.0%)	2 (13.3%)	
V	4 (13.3%)	1 (6.7%)	3 (20%)	

*ALND* Axillary lymph node dissection, *SLNB* Sentinel lymph node biopsy, *AC-T* doxorubicin/cyclophosphamide followed by docetaxel, *EC-T* Epirubicin/cyclophosphamide, *TcbHP* docetaxel/carboplatin/trastuzumab/pertuzumab

^a^Independent t-test

^b^Fisher’s exact test

^c^Mann-Whitney U test

### Feasibility results

**Fig. 1:**
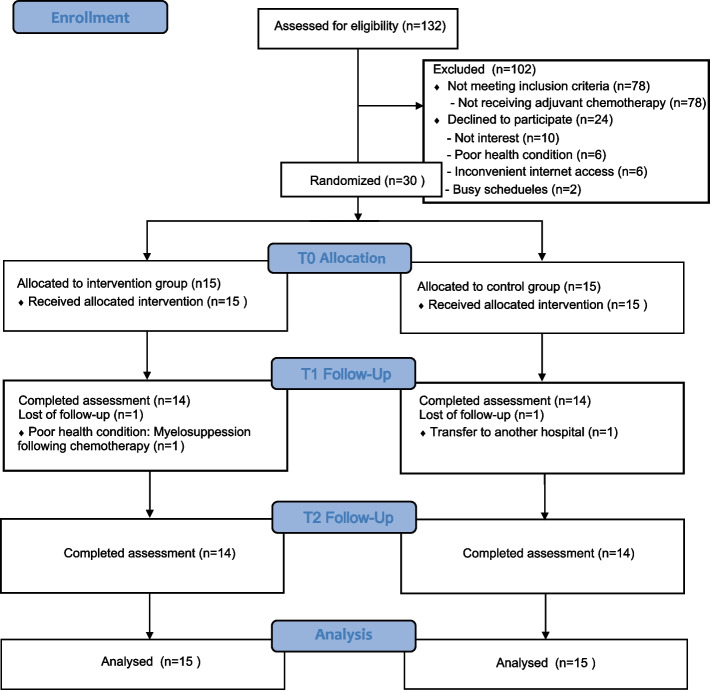
Flow diagram of this study

As shown in the study flow in Fig. 1, 132 patients were assessed for eligibility, of whom 78 participants failed to match the inclusion and exclusion criteria, resulting in an eligibility rate of 40.9%. Among the remaining 54 eligible patients, 24 declined to participate in the study for the following reasons: lack of interest (*n* = 10), poor health condition (*n* = 6), inconvenient internet access (*n* = 6), and busy schedules (*n* = 2), yielding a recruitment rate of 55.6%. All 30 participants who were recruited completed the baseline data collection. Two participants withdrew from the study, one from the intervention group due to myelosuppression following chemotherapy and one from the control group due to hospital transfer, resulting in a retention rate of 93.3%. Five of the 15 participants in the intervention group did not complete all sessions: one dropped out of the intervention group missing a total of 14 sessions, one missed eight sessions, and three missed three sessions. The adherence and session attendance rates were 66.7% and 91.4%, respectively, in the intervention group. No adverse events related to the yoga intervention were reported.

### Acceptability results

Fourteen participants in the intervention group completed the acceptability questionnaire after the intervention. Overall, 92.9% of participants expressed total satisfaction with the whole yoga intervention. All participants were able to remember the yoga movements and practice independently, and they expressed high satisfaction with the combined delivery format and practice venues. The majority of participants considered the intervention content (78.6%) and difficulty level (85.7%) to be appropriate. Three components of the yoga intervention—breathing (85.7%), postures (92.9%), and meditation (85.7%)—were rated as useful. Regarding intervention session duration, most participants thought it was appropriate, but one participant preferred shorter sessions. In open-ended questions, most participants showed their willingness to continue practicing yoga intervention after the end of the study. Three participants suggested including more full-body displays of movements in the online yoga videos used for home-based sessions (Table 2).

**Table 2:** Yoga intervention satisfaction

Item	Strongly disagree	Disagree	Neutral	Agree	Strongly agree
The content of yoga intervention is appropriate	0 (0%)	0 (0%)	0 (0%)	3 (21.4%)	11 (78.6%)
The number of sessions and duration of each sessions are appropriate	0 (0%)	0 (0%)	1 (7.1%)	3 (21.4%)	10 (71.5%)
The venue is appropriate	0 (0%)	0 (0%)	0 (0%)	0 (0%)	14 (100%)
I think that the difficulty level of yoga intervention is appropriate for me	0 (0%)	0 (0%)	0 (0%)	2 (14.3%)	12 (85.7%)
I think that the breathing exercises is useful for me	0 (0%)	0 (0%)	0 (0%)	2 (14.3%)	12 (85.7%)
I think that the yoga postures are useful for me	0 (0%)	0 (0%)	0 (0%)	1 (7.1%)	13 (92.9%)
I think the meditation is useful for me	0 (0%)	0 (0%)	0 (0%)	2 (14.3%)	12 (85.7%)
I am satisfied with the in-person sessions and home-based sessions delivery method	0 (0%)	0 (0%)	0 (0%)	0 (0%)	14 (100%)
I can remember the contents and practice yoga intervention on my own, without the assistance of online videos and supervision	0 (0%)	0 (0%)	0 (0%)	0 (0%)	14 (100%)
Overall, I am satisfied with the content and design of whole yoga intervention	0 (0%)	0 (0%)	0 (0%)	1 (7.1%)	13 (92.9%)

### Preliminary effects of the intervention

Statistically significant interaction effects were observed in the severity of the symptom cluster (*β* = −0.77; 95% confidence interval [CI] = −1.51, −0.03; *P* = 0.042; Hedge’s g = −0.313), depression (*β* = −1.27; 95%CI = −3.09, −0.06; *P* = 0.045; Hedge’s g = −0.310), and health-related quality of life (*β* = 13.46; 95%CI = 3.88, 23.05; *P* = 0.006; Hedge’s g = 0.327) at T1. The remaining outcome results were nonsignificant (Table 3).

**Table 3:** Results of outcome variables in the study groups across time using GEE model

Outcomes	Times	Intervention group Mean (SD) (*n* = 15)	Control group Mean (SD) (*n* = 15)	Group effect		Time effect		Group*Time effect		Hedge’ g
				*β* (95%CI)	*P*	*β* (95%CI)	*P*	*β* (95%CI)	*P*	*P*
Symptom cluster severity	T0	2.80 (0.97)	2.97 (0.92)	−0.13 (−0.77,0.52)	0.102					
	T1	3.34 (0.79)	3.63 (1.00)			1.38 (0.91, 1.86)	0.001*	−0.77 (−1.51, −0.03)	0.042*	−0.313
	T2	3.56 (1.20)	3.87 (0.94)			0.75 (0.48,1.01)	0.001*	−0.30 (−0.72, 0.11)	0.152	−0.280
Symptom cluster interference	T0	3.16 (0.79)	3.13 (0.82)	−0.03 (−0.53, 0.59)	0.061					
	T1	3.51 (0.95)	3.68 (1.02)			1.06 (0.70, 1.42)	0.001*	−0.90 (−1.26, 0.33)	0.082	−0.168
	T2	3.71 (1.01)	3.95 (0.90)			1.42 (0.89, 1.97)	0.039*	−0.67 (−1.50, 0.23)	0.107	−0.244
Anxiety	T0	5.87 (2.45)	5.87 (2.64)	−0.91 (−1.79, 0.02)	0.075					
	T1	5.73 (2.12)	6.33 (3.11)			0.47 (−1.08, 2.01)	0.545	−0.60 (−2.46, 1.26)	0.528	−0.216
	T2	6.03 (2.68)	7.38 (3.93)			2.33 (−0.21, 4.89)	0.073	−2.67 (−5.31, 0.78)	0.144	−0.391
Depression	T0	4.80 (2.86)	5.06 (2.63)	−0.267 (−2.17, 1.63)	0.292					
	T1	6.27 (4.13)	7.80 (5.40)			2.73 (−0.23, 4.24)	0.102	−1.27 (−3.09, −0.06)	0.045*	−0.310
	T2	6.46 (5.10)	9.53 (6.64)			4.47 (−1.45, 7.34)	0.221	−2.80 (−5.54, 0.06)	0.173	−0.505
Stress	T0	8.59 (5.28)	9.07 (5.95)	0.20 (4.64, 9.49)	0.324					
	T1	8.93 (5.04)	9.86 (5.71)			1.80 (−1.23, 4.83)	0.244	−2.13 (−5.53, 1.27)	0.219	−0.168
	T2	9.33 (5.34)	10.04 (5.54)			2.33 (−1.27, 5.94)	0.205	−3.07 (−7.22, 1.09)	0.148	−0.127
Function status	T0	75.33 (8.34)	72.67 (10.99)	2.67 (−4.08, 9.41)	0.439					
	T1	68.00 (8.61)	65.33 (5.16)			−7.33 (−12.71, −1.96)	0.008*	−0.23 (−6.65, 6.04)	0.631	0.366
	T2	64.47 (8.33)	63.66 (7.24)			−8.00 (−15.43, −0.56)	0.035*	−0.67 (−9.54, 8.20)	0.883	0.101
Health-related quality of life	T0	98.33 (16.13)	97.07 (17.85)	1.27 (−10.50, 13.03)	0.833					
	T1	96.00 (12.23)	91.26 (15.74)			−5.80 (−11.25, −0.36)	0.037*	13.46 (3.88, 23.05)	0.006*	0.327
	T2	92.80 (16.26)	87.67 (15.74)			−9.40 (−19.32, 0.52)	0.063	18.86 (−0.35, 34.38)	0.107	0.312

*p < 0.05

## Discussion

This pilot study indicated that the yoga intervention was feasible and acceptable for breast cancer patients receiving adjuvant chemotherapy, showing a high retention rate (93.3%), attendance rate (91.4%), and satisfaction rate (92.9%). Moreover, this study provided preliminary evidence that yoga intervention had positive effects on managing the fatigue-pain-sleep disturbance symptom cluster, alleviating depression, and improving health-related quality of life in this population.

The strengths of this study were reflected in the higher retention rate and attendance rate of intervention sessions compared with previous studies [34, 35]. Chemotherapy is cyclical in nature and requires patients to return to the hospital for treatment regularly. Between treatment cycles, patients typically return home to rest and await the next chemotherapy session. Based on such situation and previous evidence, this study adopted a delivery model combining in-person sessions and home-based sessions. This mode provided professional guidance and timely feedback, thereby enhancing the flexibility of the intervention [36, 37]. In particular, for the high-frequency long-term intervention in this study, this delivery mode minimized interference from weather and traffic, increased the acceptability of the intervention and the feasibility of participation, and resulted in high satisfaction from participants.

However, this delivery mode presents specific requirements for implementing the yoga intervention. During the home-based sessions, online yoga videos were used to guide the participants. These videos included numerous close-up shots to clearly show specific movements. However, participants expressed a preference for full-body displays rather than close-up views. Participants reported that focusing on specific body parts made it easy for them to lose control of other areas, making it difficult to maintain overall body awareness and coordination during yoga practice. Yoga emphasizes holistic balance and mind–body connection, encouraging practitioners to observe and feel their bodies throughout movement and stillness [22]. Therefore, it is crucial for the instructors to emphasize overall coordination and help participants practice correctly, particularly in yoga interventions delivered through online platforms.

In this pilot study, the recruitment rate was 55.6%, which was lower than that reported in similar randomized controlled trials of yoga interventions among breast cancer patients [38, 39]. This is likely because the patients were unfamiliar with what yoga was. During the recruitment process, many eligible patients declined to join the study due to a lack of interest (*n* = 10). Although all patients had heard of yoga, most were uncertain about what it actually entailed, had never practiced it, and did not understand how yoga could benefit them. The specialized nature of yoga and potential additional costs (e.g., classes and clothing) may discourage some individuals from participating [40]. Additionally, insufficient information support for symptom management during disease treatment leads to patient uncertainty about beneficial symptom management strategies [41]. This uncertainty can hinder patients’ choices and may reduce the study’s recruitment rate. Therefore, providing relevant information before the recruitment process and offering free intervention may enhance potential participants’ awareness and willingness to participate, thereby improving recruitment rates.

This pilot study also obtained a lower adherence rate than the other yoga intervention studies for breast cancer patients [42, 43]. The possible explanation is that the intervention in this study was frequent and long-term compared with others. Although such an intervention design may benefit symptom cluster management, it could negatively influence adherence. On the one hand, frequent interventions can easily cause patients to forget practice schedules, leading to missed sessions. Reminders from researchers were necessary to help participants adhere to the schedule and complete the intervention as planned. However, if reminders are too frequent and unstructured, it may not only fail to improve the intervention adherence rate but also cause information overload for participants [44]. On the other hand, long-term and frequent intervention is more likely to conflict with the patient’s daily activities and treatment plans, leading to intervention absences. Providing patients with multiple intervention schedules may increase the flexibility of intervention implementation and improve adherence. Furthermore, this study did not report any adverse events related to the yoga intervention. A possible reason is that the yoga intervention was specifically tailored to the target population, with careful consideration of participants’ physiological conditions and functional limitations. The movements were designed to be gentle and safe, which may have minimized the risk of adverse events. Meanwhile, the intervention was conducted under the close supervision of qualified yoga instructors, which further ensured its safety. These findings suggest that the yoga intervention developed in this study provides a sound foundation for safe application among a broader population of breast cancer patients.

In this study, the symptom cluster outcome was measured as a composite score, reflecting the overall effect of multiple co-occurring symptoms. This approach differs from that used in previous symptom cluster studies [45, 46], which primarily assessed individual symptoms within the cluster and inferred the symptom cluster outcomes based on differences in these individual symptoms. Such an approach assumes that the symptom cluster is significantly affected only when all the symptoms within the cluster change significantly. However, this assumption may limit the comprehensive evaluation of intervention effectiveness. Specifically, it may fail to capture clinically meaningful improvements when most, but not all, symptoms improve, even though this may still represent a meaningful relief of the symptom cluster. Under such circumstances, evaluating effectiveness solely based on significant improvement across all symptoms may underestimate the actual intervention effect. Moreover, this approach emphasizes changes in individual symptoms and might ignore the interrelationships and synergistic effects among symptoms, which are particularly crucial in managing the symptom cluster. In contrast, the composite score used in this study may better capture the overall changes in the symptom cluster but may also, to some extent, overlook the specific variations of individual symptoms within the cluster. Therefore, future studies should employ validated measurement tools that assess both overall changes in the symptom cluster and variations among individual symptoms within the cluster, providing a more comprehensive understanding of the intervention’s effects and its implications for symptom management.

This study yielded preliminary evidence supporting the management of the fatigue–pain–sleep disturbance symptom cluster. This effect may be attributed to yoga’s influence on multiple biological pathways, including the regulation of inflammatory mediators and neuroendocrine responses [47], thereby reducing the severity of the symptom cluster. Consistent with previous studies, depression levels and health-related quality of life were also improved [48, 49]. Breast cancer patients often face anxiety, depression, and other negative emotions due to cancer diagnosis, surgery, chemotherapy side effects, hair loss, and other physical damages, which can adversely affect their overall survival outcomes [50]. Yoga is a comprehensive mind–body intervention encompassing both physical and psychological components, which may help breast cancer patients regulate autonomic function, alleviate stress, and reduce anxiety and depressive symptoms [51]. However, this pilot study did not report significant results for anxiety like previous studies’ findings, possibly due to the small sample size of this study. Nevertheless, lower anxiety scores were observed in the intervention group compared with the control group.

There are some limitations to this pilot study. First, this study was conducted in only one hospital, limiting the generalizability of the findings. Second, the small sample size might weaken the statistical power of the outcome results. Third, participants were followed up for only one month subsequent to the intervention, with a lack of long-term follow-up data. Given the long period of chemotherapy and the tendency for the fatigue-pain-sleep disturbance symptom cluster to persist after chemotherapy completion [3], extending follow-up would help to evaluate the long-term effects of yoga intervention. Nevertheless, this pilot study demonstrated the available feasibility and acceptability of yoga in breast cancer patients receiving adjuvant chemotherapy and yielded preliminary positive results. Therefore, full-scale randomized controlled trials with prolonged follow-up are warranted to further evaluate the effectiveness of yoga intervention.

## Conclusion

This pilot study suggests that yoga intervention is feasible and acceptable for breast cancer patients receiving adjuvant chemotherapy and exhibits beneficial impacts on fatigue-pain-sleep disturbance symptom cluster management, depression relief, and health-related quality of life improvement. Future large-scale randomized controlled trials are warranted to further evaluate the effectiveness of yoga intervention in a larger population and over an extended follow-up period.

## Supplementary Information

Supplementary Material 1.

Supplementary Material 2.

## Data Availability

The datasets used and analyzed during the current study are available from the corresponding authors on reasonable request.
