# Supplementary material for: Yoga intervention on the fatigue-pain-sleep disturbance symptom cluster for breast cancer patients receiving adjuvant chemotherapy: a pilot randomized controlled trial
Source: BMC Complement Med Ther. 2026 Feb 10;26:99. doi: 10.1186/s12906-026-05285-7 (PMC12990628; doi:10.1186/s12906-026-05285-7)
Supplement: Supplementary file 2 — Supplementary Material 2. [file 12906_2026_5285_MOESM2_ESM.docx]

**Yoga intervention satisfaction**

| Item | Strongly disagree | Disagree | Neutral | Agree | Strongly agree |
| --- | --- | --- | --- | --- | --- |
| 1. The content of the yoga intervention is appropriate. |  |  |  |  |  |
| 1. The number of sessions and duration of each session are appropriate |  |  |  |  |  |
| 1. The venue is appropriate. |  |  |  |  |  |
| 1. I think that the difficulty level of the yoga intervention is appropriate for me |  |  |  |  |  |
| 1. I think that breathing is useful for me. |  |  |  |  |  |
| 1. I think that the yoga postures are useful for me |  |  |  |  |  |
| 1. I think the meditation is useful for me |  |  |  |  |  |
| 1. I am satisfied with the in-person sessions and home-based sessions delivery method |  |  |  |  |  |
| 1. I can remember the contents and practice yoga intervention on my own, without the assistance of online videos. |  |  |  |  |  |
| 1. Overall, I am satisfied with the content and design of the whole yoga intervention |  |  |  |  |  |

**Socio-demographic Sheet**

Date: _________ Hospital number: ___________ Interview number: _________

1. Name：_________
2. Age: _________
3. Marital Status: ① Married ② Unmarried ③ Divorced ④ Widowed
4. Residence: ① Rural ② Urban
5. Mode of residence: ① Living alone ② Living with spouse or children ③ Other
6. Education: ① Primary school ② Junior high school ③ Senior high school/Technical secondary school ④ Bachelor’s degree and above
7. Employment status: ① On the job ② On sick leave ③ Unemployed ④ Retired
8. Precipitate monthly household income: ① **<**3000 RMB ② 3000-5999 RMB

③ 6000-8999 RMB ④ **≥**9000 RMB

1. Payment method for medical expenses: ① Self-funded ② Medical insurance
2. Cancer stage: ① Ⅰ ② Ⅱ ③ Ⅲ
3. Surgery: ①Breast-conserving surgery ②Modified radical mastectomy
4. ALND (axillary lymph node dissection): ① Yes ② No
5. SLNB (sentinel lymph node biopsy): ① Yes ② No
6. Chemotherapy regimens: ________________
7. Current chemotherapy cycle: __________________
